# Water Desalination Using Polyelectrolyte Hydrogel: Gibbs Ensemble Modeling

**DOI:** 10.3390/gels8100656

**Published:** 2022-10-15

**Authors:** Mikhail Laktionov, Lucie Nová, Oleg V. Rud

**Affiliations:** 1 Department of Physical and Macromolecular Chemistry, Faculty of Science, Charles University in Prague, 12800 Prague, Czech Republic; 2 Saint-Petersburg National Research University of Information Technologies, Mechanics and Optics, 197101 Saint-Petersburg, Russia; 3 Institute of Macromolecular Compounds of Russian Academy of Sciences, 199004 Saint-Petersburg, Russia

**Keywords:** polyelectrolye hydrogel, simulation, desalination

## Abstract

Polyelectrolyte hydrogels can absorb a large amount of water across an osmotic membrane as a result of their swelling pressure. On the other hand, the insoluble cross-linked hydrogel network enables dewatering under the influence of external (thermal and/or mechanical) stimuli. Moreover, from a thermodynamic perspective, a polyelectrolyte hydrogel is already an osmotic membrane. These properties designate hydrogels as excellent candidates for use in desalination, at the same time avoiding the use of expensive membranes. In this article, we present our recent theoretical study of polyelectrolyte hydrogel usage for water desalination. Employing a coarse-grained model and the Gibbs ensemble, we modeled the thermodynamic equilibrium between the coexisting gel phase and the supernate aqueous salt solution phase. We performed a sequence of step-by-step hydrogel swellings and compressions in *open* and *closed* systems, i.e., in equilibrium with a large and with a comparably small reservoir of aqueous solution. The swelling in an *open system* removes ions from the large reservoir, whereas the compression in a *closed system* decreases the salt concentration in the small reservoir. We modeled this stepwise process of continuous decrease of water salinity from seawater up to freshwater concentrations and estimated the energy cost of the process to be comparable to that of reverse osmosis.

## 1. Introduction

Wastewater treatment and technology are one of the greatest concerns of modern society and must dispose of both biological [1] and chemical [2,3] pollutants. Most importantly, water treatment technologies are needed for the ever-increasing demand for the production of potable water from brine, i.e., for desalination.

### 1.1. Water Desalination Technologies

Two basic approaches for separating water from salt are present in modern desalination technology [4,5].

The first approach is distillation, which uses heat to cause a phase change of the water to vapor. The vapor phase is separated from the brine and condenses to liquid fresh water. The released condensation energy is directed back to heat the feed solution. Distillation was the first desalination technique conducted on a large commercial scale and still accounts for a large portion of the modern world’s desalination capacity.

The second approach is to physically separate the brine components using an osmotic membrane through which only water molecules can pass; the water molecules move in response to the difference in water chemical potential. In the context of our study, we mention reverse osmosis (RO) as the major process of all modern desalination industries, and the newly emerging membrane technology is described as forward osmosis (FO) [6]. In RO, the difference in water chemical potential originates from a difference in pressures applied to the feed and product solutions. In FO, the chemical potential difference is due to an addition to the solution from one side of the membrane (draw solution)—the so-called draw solutes, which lower the water chemical potential in the draw solution.

Distillation is easy and cheap technology, but it is characterized by relatively high energy costs due to the dissipation of thermal energy. In turn, RO uses expensive osmotic membranes that need to be replaced regularly because of scaling and fouling. Moreover, RO requires very high operating pressures, ranging from 20 to 200 bar, to let the water pass through the membrane. However, in terms of energy losses, it works close to the thermodynamic limit. Thus, theoretically, for each ion pair transferred from freshwater to saltwater, RO consumes only the energy equal to the difference between the chemical potentials of the transferred ions.

The absence of large hydraulic pressure in FO (unlike in RO) reduces the energy consumption in pumping and decreases membrane scaling and fouling, therefore significantly increasing the lifetime of the membranes. In FO, the draw solutes (agents) are dispersed and/or dissolved in water to form homogeneous draw solutions. The correct choice of draw agents is of paramount importance. As an osmotically driven process, the draw solute is expected to significantly reduce the water chemical potential and consequently generate high osmotic pressure. On the other hand, the draw solute is expected to be easily separated from water [7].

### 1.2. Hydrogels for Desalination

Hydrogels are three-dimensional networks of polymer chains that are crosslinked by either physical or chemical bonds. They can entrap large volumes of water that are attracted by the high concentration of hydrophilic groups. When a dehydrated or deswollen hydrogel uptakes water, its polymer chains extend, creating swelling pressure. For example, as reported in [8], weakly crosslinked poly(acrylic acid) (PAA) copolymers with polymer volume fractions between 0.03 and 0.30 exhibit swelling pressure ranging from 0.20–4.23 MPa. *Polyelectrolyte* hydrogels, which carry ionic groups on the comonomer units (similar to PAA), can reject salt ions from the solution, i.e., they absorb a solution of lower salinity than that of the solution with which they are equilibrated.

An important advantageous aspect of polymer hydrogels is that they can undergo reversible volume change, i.e., gel–solution volume phase transitions in response to external stimuli. This aspect causes hydrogels to be labeled as ‘smart’ materials. Many physical and chemical stimuli have been applied to induce various responses in such smart hydrogels, in particular, to change them from hydrophilic to hydrophobic, thereby releasing water. The physical stimuli include: temperature, solvent composition, light, mechanical pressure, sound, and electric and/or magnetic fields, whilst the chemical (or biochemical) stimuli include pH, ionic strength, and specific molecular recognition [9,10,11,12].

Li et al. [13] took advantage of the use of smart hydrogels for desalination purposes as draw agents for FO. They demonstrated that hydrogels are able to absorb water across the FO membrane due to their swelling and osmotic pressure and allow dewatering under the influence of stimuli (thermal and/or mechanical) due to their insoluble cross-linked polymer network. Li et al. proposed the use of hydrogels based on a thermoresponsive polyelectrolyte—a copolymer of poly-N-isopropyl acrylamide (p-NIPAAm) and polyacrylic acid (p-AA). Depending on the temperature, this gel network is either hydrophilic or hydrophobic, so it accumulates water inside the network in its hydrophilic state, but it releases water in its hydrophobic state.

From a thermodynamics perspective, the polyelectrolyte hydrogel itself is an osmotic membrane that generates a Donnan potential, which rejects ions between outer and inner solutions [14]. Such a view of hydrogels was employed in a series of works by the group of Prof. Wilhelm (see, for example, [15,16]). The authors of these works proposed to get rid of the osmotic membrane and simply use only microfiltration to compress the hydrogel, squeezing out the accumulated water inside the gel solution. In their method, the deswollen hydrogel was first equilibrated with a saline water feed. During equilibration, the gel swelled, absorbing water. Then, the gel was taken out of the feed solution and mechanically squeezed by means of a microfiltration membrane. The squeezed-out brine was found to have lower salinity than the feedwater.

A similar approach was used by Ali et al. [17]. Here, the authors used a thermosensitive gel (based on copolymers p-NIPAAm and p-AA), and instead of physical compression, dewatering was done by external heating (sunlight). The gel was equilibrated with feedwater during the night, and in the daytime under sunlight, the gel shrank, releasing the solution.

### 1.3. Physics behind the Desalination Process

Since polyelectrolyte gels have charges and neutralizing counterions, the density of mobile ions (which can freely enter and leave the gel) inside the gel network appears to be lower than their density outside the gel. Therefore, the internal solution in the gel has a lower density of mobile ions than the solution outside. In that sense, the gel acts as an osmotic membrane, separating solutions [18]. The driving force of the separation is the Donnan potential, which originates from the charges in the hydrogel network. The difference between the densities (concentrations) of the mobile ions in the internal and external solutions is defined by Donnan’s law [19]

(1) $$\frac{c_{{\mathrm{Cl}}^{-}}^{\mathrm{gel}}}{c_{s}}=\frac{c_{s}}{c_{{\mathrm{Na}}^{+}}^{\mathrm{gel}}}=\sqrt{1+{\left(\frac{\alpha }{2c_{s}v_{\mathrm{gel}}}\right)}^{2}}\pm \frac{\alpha }{2c_{s}v_{\mathrm{gel}}}$$

where $c_{{\mathrm{Cl}}^{-}}^{\mathrm{gel}}$ and $c_{{\mathrm{Na}}^{+}}^{\mathrm{gel}}$ are the concentrations of monovalent anions and cations, respectively, in the internal solution, $c_{s}$ is the salinity of the external solution, $v_{\mathrm{gel}}$ is the gel molar volume (inverse density of gel segments), and α is the ionization degree (for our study, α=1). The “±” sign in the formula accounts for the sign change in polyanion vs. polycation gels.

As shown in [13,15,16,17], a solution of lower salinity can be extracted from the gel by means of compression and/or other stimuli provided that the charge in the gel remains constant. In the case of weak polyelectrolyte (pH-sensitive) gels, compression discharges the gel, and therefore, the neutralizing counterions leave the gel, diminishing the desalination effect [19].

One can argue that the Donnan effect alone is insufficient to achieve high salt rejection [7], and the salinity of the water squeezed from hydrogels under very high hydraulic pressure (up to 100 bar [16]) turns out to be not much different from the initial salinity. Indeed, the use of high hydraulic pressure diminishes all the advantages of this method over RO, and the reversibility of hydrogel swelling after strong compression remains questionable. Nevertheless, in this study, we limit ourselves to low compression rates, less than 5 bar, when modeling the compression of the gel, and we study how compression of the gel affects the surrounding salinity. We model the desalination process as a cascade of step-by-step gel swellings and compressions, driving the salinity of the supernate down to potable water.

## 2. Results and Discussion

### 2.1. Open and Closed Systems

**Figure 1:**
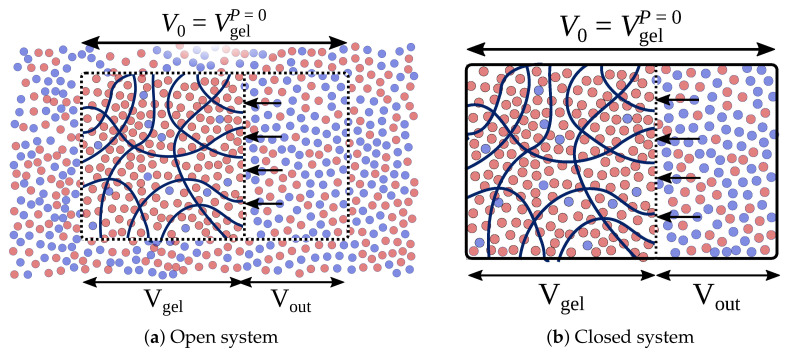
The hydrogel compressed in *open* and in *closed* system. Red and blue circles represent ${\mathrm{Na}}^{+}$ and ${\mathrm{Cl}}^{-}$ ions. $V_{0}$ is the volume which gel has in free swelling equilibrium state.

We propose the desalination process as a cascade of gel compressions and decompressions, lowering the supernatant salinity. In this process, the gel is supposed to be compressed/decompressed in an *open system* and in a *closed system*. The compression in the *open system* assumes that the gel is in thermodynamic equilibrium with a huge (effectively **infinite**) reservoir of aqueous solution Figure 1a, whereas the *closed system* implies that the gel is in equilibrium with a **finite** reservoir of an aqueous solution Figure 1b.

By thermodynamic equilibrium, we assume that the gel freely exchanges ions with the reservoir. Thus, the *open system* implies a grand canonical ensemble of mobile ions in which the change of free energy due to ion exchange is accounted for by their chemical potential. The *closed system* is the Gibbs ensemble of ions moving between two volumes of the gel phase and the supernatant.

Due to the huge size of the reservoir, compression of the gel in the *open system* does not affect reservoir salinity, $c_{s}=$ $c_{{\mathrm{Na}}^{+}}=$ $c_{{\mathrm{Cl}}^{-}}=$ Const, whereas the number of ions entrapped in the volume $V_{0}$ changes (Figure 1a). On the contrary, compression of the gel in the *closed system* changes the salinity in the reservoir, but the total number of ${\mathrm{Na}}^{+}$ and ${\mathrm{Cl}}^{-}$ ions in the gel and in the reservoir, i.e., in the volume $V_{0}$ (Figure 1b), remains constant.

Mechanical movement and the exchange of ions occur simultaneously in reality; however, we simulate them as alternating in a stop–run mode. To sample mechanical properties of the gel and reservoir, we use Molecular Dynamics (MD) simulation, whereas to sample the ion distribution between the gel and a reservoir, we use Monte Carlo (MC) simulation. The details of this hybrid MCMD computational technique can be found in our previous studies of polyelectrolytes in open systems [18,20,21] and in Section 4.

Using the data obtained from simulations, we calculate $c_{s}$, the density of ions in the outside volume; $v_{\mathrm{gel}}$, molar volume of the gel, i.e., the volume of the gel per one mol of gel segments, $v_{\mathrm{gel}}=V_{\mathrm{gel}}/N_{\mathrm{gel}}$; $n_{{\mathrm{Cl}}^{-}}$, the total number of ions in both volumes divided by the total volume of both boxes, $n_{{\mathrm{Cl}}^{-}}=N_{{\mathrm{Cl}}^{-}}/V_{0}$; and Π, *partial pressure* of the gel, i.e., the pressure that needs to be applied to the gel via a solvent-permeable filter to compress the gel to a specific molar volume. We obtain the gel partial pressure as the difference between the pressure in the gel and the pressure in the outer volume, $\Pi =P_{\mathrm{gel}}-P_{\mathrm{out}}$.

The volume $V_{0}$ was chosen to be close to the gel free-swelling equilibrium, that is, to the state where Π=0. In order to obtain the value $V_{0}$, we perform a set of open system simulations for various $V_{\mathrm{gel}}$ values. The value of $V_{\mathrm{gel}}$ at which Π is closest to zero is chosen as $V_{0}$. Then, as soon as $V_{0}$ is defined, we compress the gel in the *closed system* with varying values of $V_{\mathrm{gel}}<V_{0}$ and $V_{\mathrm{out}}=V_{0}-V_{\mathrm{gel}}$.

### 2.2. Compression in Open System

Initially, we run a set of simulations modeling the gel compression in an *open system*, i.e., in equilibrium with a big bath of certain salinity, $c_{s}$. The simulations are run for a set of different gel volumes, $V_{\mathrm{gel}}$. Each simulation returns the averaged values of pressures, $P_{\mathrm{gel}}$, and the number of ${\mathrm{Cl}}^{-}$ ions present in the simulation box, $N_{{\mathrm{Cl}}^{-}}^{\mathrm{gel}}$.

**Figure 2:**
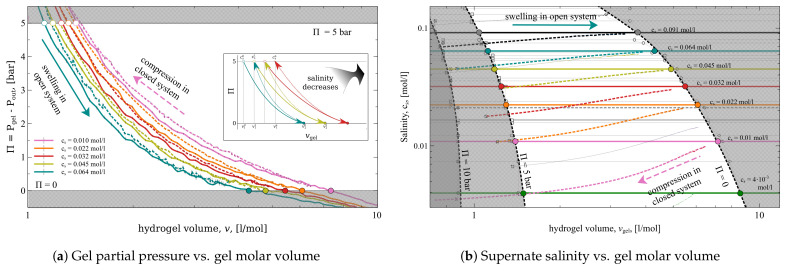
Compression of the gel in the *open system* (solid lines) and in the *closed system* (dotted lines). Each solid curve corresponds to different salinity of the reservoir $c_{s}$ (see legend). The shaded areas limit the states with applied pressure below zero and above 5 bar.

The dependencies of Π on $V_{\mathrm{gel}}$ for the *open system* for a set of various salinities are presented in Figure 2a as solid lines. For example, the blue solid line illustrates the compression (or swelling) of the gel in equilibrium with a reservoir of salinity $c_{s}=0.063$ mol/L. The points where the pressure equals zero, Π=0, (indicated by filled circles) are the gel *free-swelling equilibrium* states. These states are characterized by the corresponding molar volume of the gel, $V_{\mathrm{gel}}^{0}$, and the amount of ions in gel {$N_{{\mathrm{Na}}^{+}}^{0}$, $N_{{\mathrm{Cl}}^{-}}^{0}$} (Index “0” stands for zero bar applied pressure). The *free-swelling equilibrium* state positions shift towards smaller volumes with increased salinity. In general, increased salinity shifts all the Π(V) curves towards smaller volumes. This effect is well known and is typical for all branched *strong* polyelectrolytes. It is caused by the decrease of ion osmotic pressure and by the screening of electrostatic interactions [21,22]. (The salinity dependence on the size of a *weak* polyelectrolyte gel is in general non-monotonic. We discuss this in [19]).

### 2.3. Compression in Closed System

The simulations in the *closed systems* start from the $V_{\mathrm{gel}}^{0}$ and {$N_{{\mathrm{Na}}^{+}}^{0}$, $N_{{\mathrm{Cl}}^{-}}^{0}$} values obtained from the corresponding *open system* simulations. The simulation of gel compression in a closed volume, $V_{0}$, starts at the point $V_{0}=V_{\mathrm{gel}}^{0}$ and ion content $N_{{\mathrm{Na}}^{+}}^{0}$ for ${\mathrm{Cl}}^{-}$ ions. The number of ${\mathrm{Na}}^{+}$ ions neutralizes the system, $N_{{\mathrm{Na}}^{+}}^{0}=$ $N_{{\mathrm{Cl}}^{-}}^{0}+N_{\mathrm{gel}}$.

We prepare two systems: one for simulation of the gel at the volume $V_{\mathrm{gel}}$ and the other for simulation of the supernatant solution at the volume $V_{\mathrm{out}}=V_{0}-V_{\mathrm{gel}}$. Note that the number of $N_{{\mathrm{Cl}}^{-}}^{0}$ and $N_{{\mathrm{Na}}^{+}}^{0}$ ions are shared by the two volumes.

The processes of gel compression in the *closed system* is depicted in Figure 2a as dotted lines. In this plot, for example, the blue dotted line illustrates the compression of the gel equilibrated with solution with salinity $c_{s}=0.063$ mol/L at the volume at which the gel has zero pressure. The volume values $V_{\mathrm{gel}}$ and $V_{\mathrm{out}}$ are comparable in the *closed system* case. Therefore, gel compression decreases the salinity in the supernate, $c_{s}$. This dependence is illustrated in Figure 2b, where the same swelling/compression processes are displayed in different coordinates: i.e., salinity of the supernate versus the gel molar volume, $c_{s}\left(V_{\mathrm{gel}}\right)$. In these coordinates, all the open-system compressions show up as horizontal lines, which reflects the constant salinity, whereas the compressions in the closed system demonstrate the change of $c_{s}$ from $c_{s}^{0}=0.063$ at the zero-pressure Π to $c_{s}^{5}=0.045$ mol/L at $P^{\mathrm{gel}}=5$ bar (index “5” stands for 5 bar).

**Figure 3:**
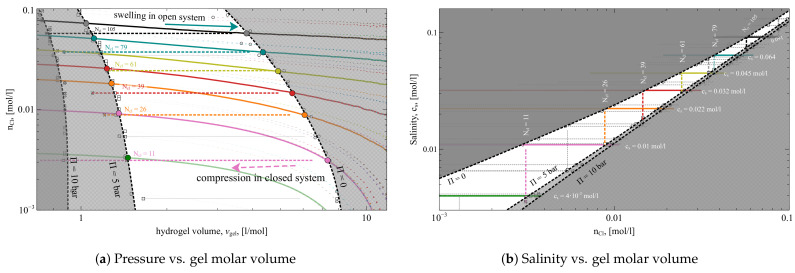
The compression of the gel in *open system* (solid lines) and in *closed system* (dotted lines). Shadowed area limit the states with applied pressure below zero and above 5 bar. The values $N_{{\mathrm{Cl}}^{-}}$ are the virtual numbers of present ${\mathrm{Cl}}^{-}$ ions in *closed system* simulation boxes.

Although the salinity during compression in the *open system* remains constant, the number of ions in the compressed subsystem (i.e., in the volume where the gel is compressed (or swells)) changes. Here, the compression volume $V_{0}$ is the volume of the *free-swelling equilibrium* state of the gel, $V_{0}=V_{\mathrm{gel}}^{0}$. Figure 3a shows number of ${\mathrm{Cl}}^{-}$ ions in the volume $V_{0}$ per unit volume, $n_{{\mathrm{Cl}}^{-}}=$ $N_{{\mathrm{Cl}}^{-}}/V_{0}$, as a function of the gel molar volume. The depicted values can be considered as the average density of ${\mathrm{Cl}}^{-}$ ions in the compression volume $V_{0}$.

The $n_{{\mathrm{Cl}}^{-}}$-$V_{\mathrm{gel}}$ dependencies look like horizontal lines in the case of *closed system* compression, whereas $n_{{\mathrm{Cl}}^{-}}$ increases with $V_{\mathrm{gel}}$ during the compression in the *open system* case. This implies, that the compression of the gel in the *open system* pulls out the ions from the bath to the compression volume, $V_{0}$. And vice versa, the swelling of the gel pushes ions out to the bath.

Finally, the same processes are depicted in Figure 3b in coordinates $n_{{\mathrm{Cl}}^{-}}$—$c_{s}$. In these coordinates, both ways of the compression, in *open* and in *closed* systems, appear as straight vertical and horizontal lines correspondingly.

In our study, we modeled the compression of the gel in equilibrium with reservoirs of 40 different salinities, ranging from 0.001 to 0.5 mol/L. The *open system* compressions resulted in defined free-swelling equilibrium states, which we used as the initial conditions for the respective compressions in the *closed system*. All the corresponding dependencies are depicted in Figure 2 and Figure 3 as thin grey dashed lines (some of them are highlighted and colored). The states corresponding to $P^{gel}=0$, 5, and 10 bar pressures are marked by open circles, squares, and crosses, respectively. The non-shaded areas in the figures highlight the states in which the gel partial pressure ranges between the experimentally relevant values of 0 and 5 bar.

### 2.4. Desalination Scheme

It follows that compression of the gel in the *closed system* affects the salinity, whereas compression in the *open system* affects the number of ions in the gel subsystem. Here, we show how to employ these phenomena for water desalination. The highlighted colored lines on the plots in Figure 2 and Figure 3 form a sequence of gel swellings and compressions, following one another and corresponding to *open* and *closed* systems. This sequence forms the water desalination process. Starting from swelling the gel in the *open system* at high salinity ($c_{s}=0.091$ mol/L, solid black line), the gel is compressed in the *closed system* until the pressure reaches 5 bar (dashed black line). Then, the same gel swells in a reservoir with slightly lower salinity in the *open system* (i.e., $c_{s}=0.064$ mol/L, light blue line). After swelling, the gel is compressed again with 5 bar pressure in the *closed system* (dashed light blue line). Then, the gel swells in a reservoir of even smaller salinity ($c_{s}=0.045$ mol/L, solid yellow line), and so on. This chain of alternating swellings and compressions ends up when salinity is equal to $c_{s}=4\times 10^{-3}$ mol/L after compression in the *closed system* (dashed magenta line).

The plots in Figure 2 and Figure 3 depict the whole process in all possible coordinate representations. In all plots, the lines corresponding to sequential swellings and compressions during the whole desalination process resemble a ’pathway’. In particular, the desalination process depicted in Figure 3b resembles a staircase, where the *open system* processes are horizontal lines and the *closed system* processes are vertical lines.

### 2.5. The Efficiency of Desalination

The theoretical minimum specific energy for seawater desalination ($c_{s}≃0.6$ mol/L for pure NaCl) is ∼3.9 kJ/L (1.1 kWh/m^3^) for 50% recovery [23]. This value is calculated as follows

(2) $$W_{\mathrm{id}}=2RT\left(\frac{c_{f}}{R_{w}}\ln\frac{c_{b}}{c_{f}}-c_{p}\ln\frac{c_{b}}{c_{p}}\right)$$

where *R* is the universal gas constant, $c_{f}$ is the salinity of the feedwater, $c_{p}$ is the salinity of the product water, $c_{b}$ is the salinity of the brine, which necessarily appears in any desalination process, and $R_{w}$ is the recovery ratio, i.e., the ratio of the volume of water produced and the feedwater volume. A 50% recovery ratio means that one part feedwater divides into two equal volume solutions of product water and brine. Of course, a significant amount of additional energy is required to operate the system [24]. It has been reported that the specific energy consumption (SEC) of reverse osmosis (RO) is 2.5–4.0 kWh/m^3^ (9.0–14.4 kJ/L), which is significantly higher than its minimum specific energy. The SEC of a real-scale RO plant is even higher, approximately 3.5–4.5 kWh/m^3^ (12.6–16.2 kJ/L), including pre-treatment and post-treatment processes [25].

To compare the efficiency of the desalination process presented in Figure 2 and Figure 3 with provided values, we collect the corresponding data in Table 1. The presented desalination process is a cascade of six swellings in an open system at six different (constant) salinities $c_{s}$, each followed by six compressions in a closed system at six different (constant) $n_{{\mathrm{Cl}}^{-}}$. Each swelling and compression process is presented as a row in Table 1, which is colored by matching the lines in the figures. The first column of the table contains values for $c_{s}^{0}$ and $c_{s}^{5}$, which stand for the supernate salinity at 0 and 5 bar compression, respectively; in an open system, supernate salinity does not change, so $c_{s}^{0}$ and $c_{s}^{5}$ are represented by a single number. The second column contains values of $n^{0}$ and $n^{5}$, which stand for the number of ${\mathrm{Cl}}^{-}$ ions in compression volume $V_{0}$ at 0 and 5 bar pressure, respectively, (divided by $V_{0}$). The number of ions does not change in closed system compression; thus, $n^{0}$ and $n^{5}$ are the same in the corresponding rows. The third column shows the change of the gel volume in the corresponding process, Δv. The fourth column contains the work needed for compression in the corresponding process per volume of extracted solution. This value is obtained as the numerical integration of corresponding $\Pi (V_{\mathrm{gel}})$ dependence [26]

(3) $$W=\frac{\int_{v^{0}}^{v^{5}}\Pi dV_{\mathrm{gel}}}{\Delta v}$$

In this column, we present the absolute values of the work, whereas one should keep in mind that compression implies that the work is done by external force, and swelling implies that the work is done by the gel.

For comparison with ideal desalination process efficiency, the fifth column provides values for ideal specific energy consumption, $W^{\mathrm{id}}$, which are calculated employing Equation (2), for the concentrations of feed, product, and brine solutions, $c_{s}^{f}$, $c_{s}^{p}$, $c_{s}^{b}$, respectively, as indicated by curly brackets. For example, with $c_{s}^{f}=44.91$, $c_{s}^{p}=31.93$, and $c_{s}^{b}=63.93$ mmol/L, (fifth, seventh, and third rows of the table), one can imagine the following desalination process

1. First, the gel equilibrates with the feed solution and is compressed in the *closed system*. The gel volume decreases by $\Delta v^{p}=3.69$ liters, and the salinity of the supernate decreases from $c_{s}^{f}$ to $c_{s}^{p}$. The volume of the product solution is $\Delta v^{p}$.
2. The squeezed gel is put back into the feed solution and is equilibrated there under pressure, so it does not swell.
3. After equilibration, the gel swells in the *closed system*, so the salinity of the external solution increases to the value $c_{s}^{b}$.
4. Finally, the gel is taken out and compressed at 5 bar pressure in the *open system* in equilibrium with the brine bath. The change of the gel volume in this process is $\Delta v^{b}=3.26$ L/mol, which equals the volume of the produced brine.

Thus, the recovery ratio $R_{w}=\Delta v^{p}/\left(\Delta v^{p}+\Delta v^{b}\right)≃$ 0.53 and the theoretical minimum specific energy of the desalination process with corresponding $c_{s}^{f}$, $c_{s}^{p},$ and $c_{s}^{b}$ is $W^{id}=$ 38.2 J/L (Equation (2)).

The estimated $W^{id}$ values are provided in the fifth column of the table for five triplets of $c_{s}^{f}$, $c_{s}^{p}$,$c_{s}^{b}$ values. In the same column, we provide $W_{\mathrm{sim}}$—the specific energy consumption calculated by a numerical integration of $\Pi (V_{\mathrm{gel}})$ dependencies. The provided values $W_{\mathrm{sim}}$ are the sum of energies needed for corresponding compression processes, i.e., in the *closed* and *open* systems. The values $R_{w}$, which are also provided in fifth column, are the corresponding recovery ratios.

The ratio between $W_{\mathrm{sim}}$ and $W_{\mathrm{id}}$ ranges from 3.26 to 5.43, which is comparable to that of RO. Note that when calculating $W_{\mathrm{sim}}$, we accounted for only the work done on the gel during compression, whereas the work done by the gel itself during swelling was not taken into account. The process that accounts for energy recovery was described in our previous studies [19,27].

### 2.6. Study Limitations

Like other simulation-based studies, our research has limited validity, primarily resulting from the simplifications applied in the used model. For example, our coarse-grained model cannot differentiate polystyrene sulphonate gel from other strong polyacidic gels. However, these limitations are also the advantage of our model, because the results of our study can be applied to similar systems, including polybasic gels with all the charges reversed.

### 2.7. Implications and Future Perspectives

We are aware that the concept introduced in this simulation study needs to be experimentally verified. Therefore, in the future, we want to focus on experimental aspects of desalination based on polyacidic gel compression.

## 3. Conclusions

We have modeled compression of a polyelectrolyte gel in thermodynamic equilibrium with a supernatant aqueous solution of limited amount. We have shown that compression of the gel decreases the supernatant salinity. We employed this phenomenon to model water desalination. The desalination was done as a sequential combination of two processes: (1) swelling of the gel in an *open system*, exchanging ions with a large reservoir at constant salinity; (2) compression of the gel in a *closed system*, during which the gel exchanges ions with a small reservoir, affecting its salinity. We estimated the energy consumption needed for producing one liter of potable water from brine and have shown that the proposed gel compression method may compete with modern desalination technologies.

## 4. Materials and Methods

### 4.1. Molecular Dynamics

**Figure 4:**
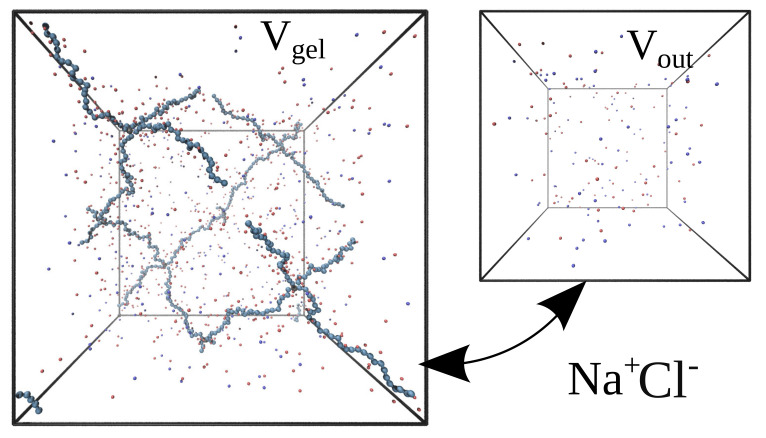
Diamond-like network in the simulation box. Red and blue particles are the ions ${\mathrm{Na}}^{+}$ and ${\mathrm{Cl}}^{-}$.

We model the gel as a network of 16 linear polymer chains, each consisting of 30 monomer units. These polymer chains are connected to a diamond-like network by eight crosslinking units. This means there are $N_{\mathrm{gel}}=16\cdot 30+8=488$ gel monomers in the simulation box (see Figure 4). The network is put in a simulated cubic box with volume $V_{\mathrm{gel}}$ with periodic boundary conditions, which virtually emulates an infinite polymer network.

Each monomer unit of the network carries a negative elementary electric charge. Except for the gel monomers, the monovalent co- and counter-ions, ${\mathrm{Cl}}^{-}$ and ${\mathrm{Na}}^{+}$, are present in the simulation box. The total electric charge of all the particles in the box is zero; therefore the number of ${\mathrm{Na}}^{+}$ ions exceeds the number of ${\mathrm{Cl}}^{-}$ ions by $N_{\mathrm{gel}}$.

Each pair of particles interact via the truncated Lennard–Jones interaction potential, which imposes strong repulsion between all particles at short distances:

(4) $$\begin{array}{c} V_{\mathrm{LJ}}\left(r\right)=\left\{\begin{array}{cc} 4\varepsilon \left({\left(\frac{\sigma }{r}\right)}^{12}-{\left(\frac{\sigma }{r}\right)}^{6}\right)\; & \mathrm{if}\;r<r_{\mathrm{cut}}\\ 0\; & \mathrm{elsewhere} \end{array}\right., \end{array}$$

where *r* is the interparticle distance, σ=0.35nm is a chosen characteristic size of the particles, $\varepsilon =k_{B}T$ is the depth of the potential, and $r_{\mathrm{cut}}$ is the cut-off distance beyond which the potential is set zero.

The bonds connecting the gel to a network are modeled using finite extension nonlinear elastic potential (FENE)

(5) $$V_{\mathrm{FENE}}\left(r\right)=-\frac{1}{2}K\Delta r_{\max}^{2}\ln\left[1-{\left(\frac{r-r_{0}}{\Delta r_{\max}}\right)}^{2}\right],$$

where *r* is the distance between the bonded segments, *K* is the magnitude of their interaction, $\Delta r_{\max}$ is the maximal stretching length of the bond, and $r_{0}$ is the equilibrium bond length. For our simulations, we chose $K=10k_{B}T/\sigma^{2}$, $\Delta r_{\max}=2\sigma$, and $r_{0}=1.0\sigma$ [28].

All the charged particles interact via Coulomb electrostatic potential:

(6) $$V_{\mathrm{EL}}=l_{B}k_{B}T\frac{q_{1}q_{2}}{r},$$

where $l_{B}$ is Bjerrum length—$l_{B}=2\sigma =0.7\;\mathrm{nm}$, which corresponds to the Bjerrum length in water at temperature T=300K—and $k_{B}$ is the Botlzman constant. In that sense, the solvent (water) is accounted for in the model implicitly via setting up dielectric permittivity ϵ=80.

We used the Langevin thermostat, i.e., two additional terms for force in the equation of motion were added

(7) $$f_{i}=-\gamma v_{i}\left(t\right)+\sqrt{2\gamma k_{B}T}\eta_{i}\left(t\right),$$

where the first term corresponds to constant friction, with γ being a friction coefficient, and the second term corresponds to random thermal force, with $\eta_{i}$ being a normally distributed random vector; $v_{i}$ is the velocity of the *i*-th particle (for details see [29]).

### 4.2. Monte Carlo Sampling in an Open System

The Monte Carlo scheme for sampling the exchange of ions in an *open system* is based on the formula for free energy of grand canonical ensemble Ω

(8) $$\Omega_{\mathrm{open}}=E-TS+\sum_{i}\mu_{i}N_{i}$$

where *E* is internal energy, *T* is temperature, *S* is entropy, $N_{i}$ is the number of ions of type $i\in \{{\mathrm{Na}}^{+},\;{\mathrm{Cl}}^{-}\}$, and $\mu_{i}$ is the corresponding chemical potential.

The entropy *S* is given by the Boltzmann formula [30]

(9) $$S=k_{B}\sum_{i}\ln\frac{V_{\mathrm{gel}}^{N_{i}}}{N_{i}!}$$

which accounts for two contributions:

1. The combinatorial entropy $S_{c}=-k_{B}\sum_{i}\ln N_{i}!$, which reflects the freedom of choice among the particles;
2. The mixing entropy $S_{m}=k_{B}\sum_{i}N_{i}\ln V_{\mathrm{gel}}$, which reflects the freedom to place the chosen particle randomly within the simulation box.

$V_{\mathrm{gel}}$ is the unitless volume, i.e., the volume measured in units of $\sigma^{3}$. Thus, the change of free energy associated with an exchange of ion pairs is

(10) $$\Delta \Omega_{\mathrm{open}}=k_{B}T\ln\prod_{i}\left(V_{\mathrm{gel}}^{\xi }\frac{N_{i}!}{\left(N_{i}+\xi \right)!}\right)+\xi \sum_{i}\mu_{i}+\Delta E$$

where ξ is an algebraic number of inserted (or removed) ion pairs; in general, ξ can be any number, but when ξ=±1, which corresponds to addition or removal of only one ion pair, Equation (10) gets simplified

(11) $$\Delta \Omega_{\mathrm{open}}=k_{B}T\ln V_{\mathrm{gel}}^{2\xi }\prod_{i}{\left(N_{i}+\theta \left(\xi \right)\right)}^{-\xi }+\xi \sum_{i}\mu_{i}+\Delta E$$

where θ is the Heaviside function; θ(ξ)=1 if ξ=+1; θ(ξ)=0 if ξ=−1.

The procedure for Monte Carlo sampling is as follows: [31]

1. Propose the new configuration of the system by insertion (or deletion) of an ion pair, ξ=±1;
2. Accept the new configuration if
  (12) $$R^{\xi }<e^{\Delta \Omega_{\mathrm{open}}/k_{B}T}=V_{\mathrm{gel}}^{2\xi }\prod_{i}{\left(N_{i}+\theta \left(\xi \right)\right)}^{-\xi }e^{\left(\Delta E+\xi \mu \right)/k_{B}T}$$
  where R is a uniformly distributed random number in the range between 0 and 1;
3. Then, collect the number of ions, $N_{{\mathrm{Na}}^{+}}$ and $N_{{\mathrm{Cl}}^{-}}$, to the samples array.

### 4.3. Monte Carlo Sampling in Closed System

In the *closed system*, the gel exchanges particles with the explicit finite reservoir box. The total number of ion species in both boxes is fixed, whereas the density of ions in the external reservoir is defined by thermodynamic equilibrium between the two subvolumes (see Figure 1b). Monte Carlo sampling of the distribution of ions between the subvolumes is performed in a way similar to that described in [32,33].

The free energy of the Gibbs ensemble is a sum of the gel’s free energy and that of the external volume.

(13) $$\Omega_{\mathrm{closed}}=E_{\mathrm{gel}}-TS_{\mathrm{gel}}+E_{\mathrm{out}}-TS_{\mathrm{out}}$$

Using reasoning similar to that of the *open system*, one can derive the change of free energy associated with ion-pair exchange

(14) $$\Delta \Omega_{\mathrm{closed}}=2k_{B}T\ln{\left(\frac{V_{\mathrm{gel}}}{V_{\mathrm{out}}}\right)}^{\xi }\prod_{i}{\left(\frac{N_{i}^{\mathrm{gel}}+\theta \left(\xi \right)}{N_{i}^{\mathrm{out}}+\theta \left(-\xi \right)}\right)}^{-\xi }+\Delta E_{\mathrm{gel}}+\Delta E_{\mathrm{out}}$$

where ξ defines the direction of the trial move, so that ξ=−1 when an ion pair moves from the gel to the outside volume, and ξ=+1 otherwise; $\Delta E_{\mathrm{gel}}$ and $\Delta E_{\mathrm{out}}$ are corresponding changes of the potential energy of the gel and the outside volumes, respectively.

Then, the procedure for sampling is the same as that of the *open system*: (1) propose the move of an ion pair; (2) accept a new state if $R^{\xi }<\exp(\Delta \Omega_{\mathrm{closed}}/k_{B}T$); and (3) repeat the procedure until the desired number of samples is reached.

### 4.4. Algorithm

As mentioned above, the whole simulation run consists of MD and MC sub-simulations of the mechanical movement of the particles and ion exchange. The algorithm is the following:

1. Initiate the systems to simulate: the gel of volume $V_{\mathrm{gel}}$ and the external solution of volume $V_{\mathrm{out}}$;
2. Equilibrate the system, interspersing the MD and MC stages;
3. Run the MD subsimulation and collect the observables: pressure in both volumes, $P_{\mathrm{gel}}$, $P_{\mathrm{out}}$, and distances between the nodes of the gel network. The latter is needed to estimate the autocorrelation of the MD simulation;
4. Run the MC procedure, simulating ion exchange, and collect the number of ions in both boxes, $N_{{\mathrm{Cl}}^{-}}^{\mathrm{gel}}$ and $N_{{\mathrm{Cl}}^{-}}^{\mathrm{out}}$;
5. Repeat the MD and MC subsimulations until the desired length of sample arrays is reached.

## Figures and Tables

**Table 1:** Estimates of desalination efficiency. All units are calculated per one mol of gel segments. Values in brackets correspond to crossection of ‘red’ and ‘orange’ lines on the plots in Figure 2 and Figure 3.

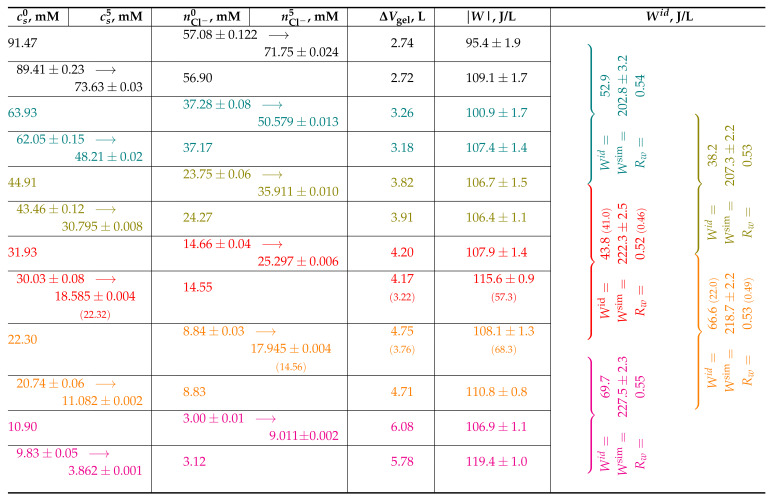

## References

[1] Guesmi A., Cherif M.M., Baaloudj O., Kenfoud H., Badawi A.K., Elfalleh W., Hamadi N.B., Khezami L., Assadi A.A. (2022). Disinfection of corona and myriad viruses in water by non-thermal plasma: A review. Environ. Sci. Pollut. Res..

[2] Baaloudj O., Badawi A.K., Kenfoud H., Benrighi Y., Hassan R., Nasrallah N., Assadi A.A. (2022). Techno-economic studies for a pilot-scale Bi12TiO20 based photocatalytic system for pharmaceutical wastewater treatment: From laboratory studies to commercial-scale applications. J. Water Process. Eng..

[3] Shahzad W., Badawi A.K., Rehan Z.A., Khan A.M., Khan R.A., Shah F., Ali S., Ismail B. (2022). Enhanced visible light photocatalytic performance of Sr0.3(Ba,Mn)0.7ZrO3 perovskites anchored on graphene oxide. Ceram. Int..

[4] Miller J. (2003). Review of Water Resources and Desalination Technologies.

[5] Curto D., Franzitta V., Guercio A. (2021). A Review of the Water Desalination Technologies. Appl. Sci..

[6] Akther N., Sodiq A., Giwa A., Daer S., Arafat H.A., Hasan S.W. (2015). Recent advancements in forward osmosis desalination: A review. Chem. Eng. J..

[7] Cai Y., Hu X.M. (2016). A critical review on draw solutes development for forward osmosis. Desalination.

[8] Wack H., Ulbricht M. (2009). Effect of synthesis composition on the swelling pressure of polymeric hydrogels. Polymer.

[9] Tanaka T., Nishio I., Sun S.T., Ueno-Nishio S. (1982). Collapse of Gels in an Electric Field. Science.

[10] Serizawa T., Wakita K., Akashi M. (2001). Rapid Deswelling of Porous Poly(N-isopropylacrylamide) Hydrogels Prepared by Incorporation of Silica Particles. Macromolecules.

[11] Lietor-Santos J.J., Sierra-Martin B., Vavrin R., Hu Z., Gasser U., Fernandez-Nieves A. (2009). Deswelling Microgel Particles Using Hydrostatic Pressure. Macromolecules.

[12] Qiu Y., Park K. (2001). Environment-sensitive hydrogels for drug delivery. Adv. Drug Deliv. Rev..

[13] Li D., Zhang X., Yao J., Simon G.P., Wang H. (2011). Stimuli-responsive polymer hydrogels as a new class of draw agent for forward osmosis desalination. Chem. Commun..

[14] Wang H., Wei J., Simon G.P. (2014). Response to Osmotic Pressure versus Swelling Pressure: Comment on “Bifunctional Polymer Hydrogel Layers As Forward Osmosis Draw Agents for Continuous Production of Fresh Water Using Solar Energy”. Environ. Sci. Technol..

[15] Arens L., Albrecht J.B., Höpfner J., Schlag K., Habicht A., Seiffert S., Wilhelm M. (2017). Energy Consumption for the Desalination of Salt Water Using Polyelectrolyte Hydrogels as the Separation Agent. Macromol. Chem. Phys..

[16] Fengler C., Arens L., Horn H., Wilhelm M. (2020). Desalination of Seawater Using Cationic Poly(acrylamide) Hydrogels and Mechanical Forces for Separation. Macromol. Mater. Eng..

[17] Ali W., Gebert B., Hennecke T., Graf K., Ulbricht M., Gutmann J.S. (2015). Design of Thermally Responsive Polymeric Hydrogels for Brackish Water Desalination: Effect of Architecture on Swelling, Deswelling, and Salt Rejection. ACS Appl. Mater. Interfaces.

[18] Rud O.V., Landsgesell J., Holm C., Košovan P. (2021). Modeling of weak polyelectrolyte hydrogels under compression – Implications for water desalination. Desalination.

[19] Rud O., Borisov O., Košovan P. (2018). Thermodynamic model for a reversible desalination cycle using weak polyelectrolyte hydrogels. Desalination.

[20] Rud O.V., Kazakov A.D., Nova L., Uhlik F. (2022). Polyelectrolyte Hydrogels as Draw Agents for Desalination of Solutions with Multivalent Ions. Macromolecules.

[21] Landsgesell J., Hebbeker P., Rud O., Lunkad R., Košovan P., Holm C. (2020). Grand-Reaction Method for Simulations of Ionization Equilibria Coupled to Ion Partitioning. Macromolecules.

[22] Zhulina E., Klein Wolterink J., Borisov O. (2000). Screening Effects in a Polyelectrolyte Brush: Self-Consistent-Field Theory. Macromolecules.

[23] Wang L., Violet C., DuChanois R.M., Elimelech M. (2020). Derivation of the Theoretical Minimum Energy of Separation of Desalination Processes. J. Chem. Educ..

[24] Kim J., Park K., Yang D.R., Hong S. (2019). A comprehensive review of energy consumption of seawater reverse osmosis desalination plants. Appl. Energy.

[25] Kim J., Hong S. (2018). A novel single-pass reverse osmosis configuration for high-purity water production and low energy consumption in seawater desalination. Desalination.

[26] Atkins P., de Paula J. (2010). Physical Chemistry.

[27] Prokacheva V.M., Rud O.V., Uhlík F., Borisov O.V. (2021). Phase transition in hydrophobic weak polyelectrolyte gel utilized for water desalination. Desalination.

[28] Jin S., Collins L.R. (2007). Dynamics of dissolved polymer chains in isotropic turbulence. New J. Phys..

[29] Grest G.S., Kremer K. (1986). Molecular dynamics simulation for polymers in the presence of a heat bath. Phys. Rev. A.

[30] Nagle J.F. (2004). Regarding the Entropy of Distinguishable Particles. J. Stat. Phys..

[31] Frenkel D., Smit B. (2002). Understanding Molecular Simulation.

[32] Panagiotopoulos A., Quirke N., Stapleton M., Tildesley D. (1988). Phase equilibria by simulation in the Gibbs ensemble. Mol. Phys..

[33] Erdos M., Galteland O., Bedeaux D., Kjelstrup S., Moultos O.A., Vlugt T.J.H. (2020). Gibbs Ensemble Monte Carlo Simulation of Fluids in Confinement: Relation between the Differential and Integral Pressures. Nanomaterials.

